# Cervical Spondylotic Myelopathy: Pathophysiology, Diagnosis, and Surgical Techniques

**DOI:** 10.5402/2011/463729

**Published:** 2011-09-28

**Authors:** Tobias A. Mattei, Carlos R. Goulart, Jeronimo B. Milano, Luis Paulo F. Dutra, Daniel R. Fasset

**Affiliations:** ^1^Department of Neurosurgery, University of Illinois College of Medicine at Peoria, Peoria, IL 6156-1649, USA; ^2^League of Neurosurgery, The Neurologival Institute of Curitiba, 81210-310 Curitiba, PR, Brazil; ^3^Instituto de Neurologia de Curitiba, 81210-310 Curitiba, PR, Brazil; ^4^Department of Neurosurgery, University of Illinois College of Medicine at Peoria, Peoria, IL, USA

## Abstract

Cervical spondylotic myelopathy is a degenerative spinal disease which may lead to significant clinical morbidity. The onset of symptoms is usually insidious, with long periods of fixed disability and episodic worsening events. Regarding the pathophysiology of CSM, the repeated injuries to the spinal cord are caused by both static and dynamic mechanical factors. The combination of these factors affects the spinal cord basically through both direct trauma and ischemia. Regarding the diagnosis, both static and dynamics X-rays, as well as magnetic resonance imaging are important for preoperative evaluation as well as individualizing surgical planning. The choice of the most appropriate technique is affected by patient's clinical condition radiologic findings, as well as surgeon's experience. In opposition to the old belief that patients presenting mild myelopathy should be treated conservatively, there has progressively been amount of evidence indicating that the clinical course of this disease is progressive deterioration and that early surgical intervention improves long-term functional recovery and neurological prognosis.

## 1. Introduction

Cervical spondylosis is the most common nontraumatic cause of myelopathy in the cervical spine [1]. Different from the majority of the other spinal problems in which the clinical treatment is usually the first option, early surgery is a key point to interfere in the natural history of cervical spondylotic myelopathy (CSM) and improve the neurological prognosis. In fact, there is strong evidence showing that surgery within one year from onset of symptoms strongly improves prognosis in CSM [1–3].

Nevertheless, the diagnosis of CSM can be difficult because the signs and symptoms can vary widely among the population. Besides, onset of symptoms is usually insidious, with long periods of fixed disability and episodic worsening events. Some findings that can commonly appear are gait spasticity, followed by upper extremity numbness and loss of fine motor control in the hands [2, 3]. 

Although it is generally agreed that surgical intervention positively impacts the prognosis of CSM, the decision algorithm for the selection of the most appropriate surgical technique is complex. In fact, the choice between a ventral or a dorsal approach depends on several factors such as the relative location of the primary compression (dorsal × ventral) and the alignment of the cervical spine (lordosis × kyphosis), as well as patient-specific spinal biomechanics [2–4].

## 2. Pathophysiology

CSM has been first defined by Brain et al. in 1952 [2]. The pathophysiology of the development of CS and subsequently CSM can be referred to as a cascade in which multiple factors play a role. The process usually begins with the degeneration of the cervical disc with further collapse of the discal space. The endplates of the vertebral bodies progressively suffer mechanical stress with the consequent formation of osteophytes. These osteophytes are a natural trial to increase the load-bearing surface of the endplates in order to compensate for spine hypermobility secondary to disk degeneration. Furthermore, ossification of the posterior longitudinal ligament (OPLL), most commonly seen in the Asian population, can also lead to contribute to CSM [5, 6].

### 2.1. Mechanical Factors

The repeated injuries to the spinal cord, which result in CSM, are caused by both static and dynamic mechanical factors. The combination of these factors affects the spinal cord basically through two mechanisms: direct trauma and ischemia [7, 8]. 

#### 2.1.1. Static Mechanical Factors

All these following factors contribute to narrowing the spinal canal

(i) the osteophyte's formation decreases the diameter of the spinal canal and may compress the spinal cord directly.

(ii) the hypertrophy of the ligamentum flavum, OPLL and subluxation, or kyphosis of the cervical spine may also serve to narrow the spinal canal.

Such static factors have a more marked impact on patients with congenital stenosis of the spinal canal [8].

#### 2.1.2. Dynamic Mechanical Factors

Dynamic stressors refer to the abnormal motion of the cervical spine during flexion or extension, which can contribute to spinal cord injury synergistically with static mechanical factors. Flexion of the cervical spine may lead to compression of the spinal cord against osteophytic bars while extension may lead to compression against the hypertrophied ligamentum flavum [1, 8, 9].

### 2.2. Ischemia

Spinal cord ischemia occurs when degenerative elements compress blood vessels that supply the cervical spinal cord and proximal nerve roots. Ischemia may result from direct compression of larger vessels such as the anterior spinal artery and overall reduced flow in the pial plexus as well as in small penetrating arteries which supply the cord [1, 10, 11]. Furthermore, impairment of venous flow may lead to significant venous congestion and contribute to spinal cord ischemia. Some postmortem studies in patients with CSM demonstrating abnormal histological findings, such as spinal cord necrosis and gray matter cavitations, have led to the conclusion that vascular mechanisms may be more involved in the pathophysiology of CSM than previously thought [1]. Furthermore, the region of the spinal cord most affected by CSM (levels C5 to C7) is also the area with the most vulnerable vascular supply [1, 9–11].

## 3. Signs and Symptoms

CSM can cause a variety of signs and symptoms. Nevertheless none of them has been proven to be pathognomonic. The onset of the disease is invariably insidious. In the initial series reported by Brain et al., the duration of symptoms ranged from one week to 26 years, and almost half of the patients presented symptoms for more than one year at the time of diagnosis [2].

In another review of 1,076 patients with CSM, gait disturbance was the most common presentation [12]. In this series, spastic gait was one of the first symptoms, followed by upper extremity numbness and loss of fine motor control of the hands. Other common symptoms of CSM are neck pain, as well as referred pain in the shoulder or subscapular area.

Furthermore, it has already been shown that one-third of patients with cervicalgia due to CSM present with headache and greater than two thirds may present with unilateral or bilateral shoulder pain. A significant number of these patients also present with irradiated pain to the arm, forearm and/or hand pain with long periods of remission [13].

Upper motor neuron findings such as spasticity, hyperreflexia, clonus, Babinski, and even bowel and bladder dysfunction may also be present. These findings often occur together with lower motor neuron findings, such as hyporreflexia and atrophy in the upper extremities. Numbness or paresthesias in the upper extremities is usually nonspecific, although dermatomal sensory complaints can occur from a coexisting radiculopathy. Sensory changes in the lower extremities is also common and typically involve the dorsal columns. Furthermore, motor weakness as well as gait impairment, are also commonly present [1, 12, 14].

## 4. Imaging Diagnosis

**Figure 1:**
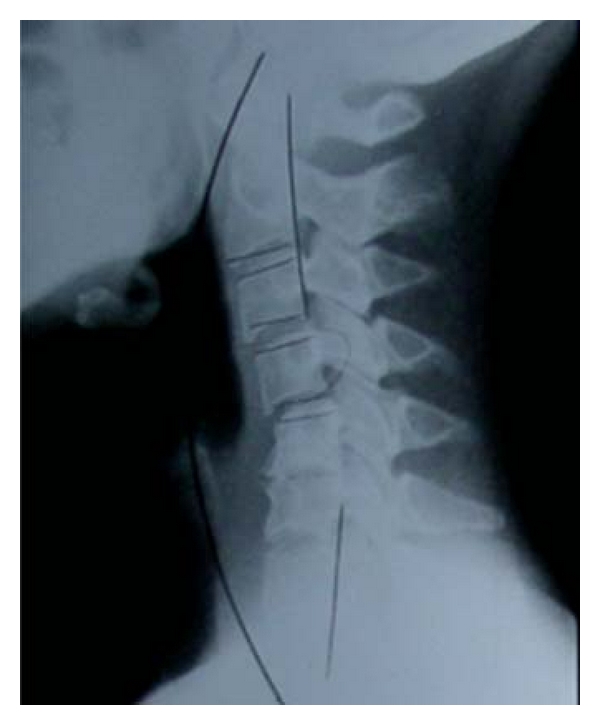
Simple radiograph demonstrating C4-C5 subluxation as well as C5-C6 degenerative spondylosis in a patient with symptoms of CSM.

The diagnostic of CSM often includes cervical radiographs, which may demonstrate osteophyte formation, kyphosis, and even subluxation (Figure 1).

**Figure 2:**
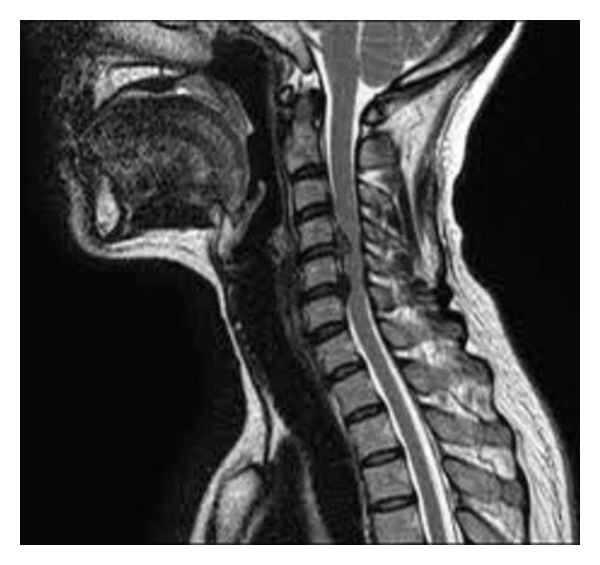
T2-weighted sagittal MRI demonstrating a 2-level CSM with predominantly anterior compression due to soft disc herniation.

Nevertheless, magnetic resonance imaging (MRI) of the cervical spine still remains the most useful diagnostic tool [7] (Figure 2). In addition to providing an evaluation of the spinal cord, the ligaments, and the intervertebral discs, MRI may also help to rule out other differential' diagnoses, such as spinal cord tumors or syrinx.

Furthermore, T2-weighted hyperintensity at the level of spinal compression has also been shown to correlate with CSM severity and has been supposed to be an important prognostic factor. Such findings are thought to represent edema and inflammation [7, 8, 15].

On the other hand T1-hypointensity has been shown to be a more severe sign, representing ischemia, myelomalacia, or gliosis as has been correlated with postoperative worst outcome [16–18].

## 5. Surgical Management

Most of the guidelines recommend operative therapy over conservative therapy for moderate to severe cases of CSM as well as for mild cases if the patient presents good clinical conditions.

The surgical management of CSM has begun with the classic anterior cervical discectomy and fusion procedure developed by Cloward and Smith [19] and Robinson [20]. Other techniques, such as posterior laminectomy and fusion procedures, as well as a vast number of laminotomy techniques have been proposed [7, 15, 19, 20]. 

The aim of the surgical procedure is to relieve spinal cord compression, as well as achieve stabilization whenever necessary. Surgical techniques can be broadly divided into anterior, posterior or combined surgical approaches. Other critical factors that must be considered in the surgical planning are the necessity to maintain or restore the alignment of the cervical spine as well as the necessity of permanent mechanical stability and fusion. Surgeons must be keenly aware of the advantages, disadvantages, and limitations of each approach [7, 8, 15, 20]. 

In summary, in relation to the selection of the best surgical approach, it is important that every patient be evaluated individually. Nevertheless, some factors, such as the sagittal balance and number of levels to be addressed may have strong influence on such choice. For example, patients with loss of cervical lordosis should not be submitted to laminectomy without fusion or laminoplasty [7, 8].

### 5.1. Anterior Surgical Techniques for CSM

When anterior compression of the spinal cord is the most important component, anterior techniques are preferred. Some examples are disc protrusions or marked osteophytosis. Anterior approaches have the advantage of more readily restoring the cervical lordosis, which is useful for cases where the kyphosis exacerbates the spinal cord compression or when loss of cervical lordosis is a contraindication for laminoplasty [21, 22]. 

Resection of the osteophyte/disc complex and placement of an interspace graft not only remove the offending ventral pathology but can also be used to restore lordosis to a straight or kyphotic spine [23–25].

Sometimes corpectomies must be added when large osteophytes extend behind the vertebral bodies. Corpectomy may also be indicated for patients with calcification of the posterior longitudinal ligament [22, 24, 25]. 

Vaccaro et al. studied the effect of the number of vertebral bodies resected on the rate of nonunion. Early instrumentation failure occurred in 9% of patients with 2-level corpectomies with bone graft and ventral instrumentation. Nevertheless the failure rates increased up to 50% in patients undergoing 3- or more level corpectomy [26–28]. 

Anterior cervical decompression and fusion (ACDF) of 1–3 levels has been reported in multiple case series to be safe and effective in decompressing ventral pathology. When performed for more than three levels or in case of more than 2 corpectomies, the rate of further complications (such as fracture, graft extrusion, and pseudoarthrosis) increases exponentially. In such cases most of the authors recommend to add further posterior instrumentation [13, 29, 30].

### 5.2. Posterior Surgical Techniques for CSM

There are mainly two posterior approaches for the treatment of CSM: laminectomy (with or without fusion) and laminoplasty.

Posterior approaches may be considered when the pathology is located at the posterior portion of the spinal canal, for example, in cases of hypertrophied ligamentum flavum. Nevertheless, posterior decompression also addresses anterior compression because it indirectly decompresses the spinal cord by enlarging the spinal canal. When compared to anterior approaches, posterior procedures offer several advantages for the treatment of CSM. Some of these factors are that they may not require fusion of that vertebral level and it enables direct visualization of the spinal canal and wide decompression of spinal cord and nerve roots.

However, some of these procedures, such as laminectomy without fusion and laminoplasty, also present some disadvantages such as development of instability or postlaminectomy kyphosis. Furthermore, none of the posterior approaches enable primary resection of anterior pathology.

#### 5.2.1. Laminectomy (with and without Fusion)

The oldest technique for posterior decompression of CSM is laminectomy without fusion. Nevertheless, the major postoperative complication of such approach is postlaminectomy instability. The groups of patients in risk for such complication are those who present signs of preexisting instability and those in which aggressive facet resection is performed. In these cases instrumentation stabilization at the time of laminectomy is recommended [7, 31]. 

Instrumented fusion serves to both stabilize the cervical spine as well as secure the spine in an optimal lordotic configuration. In relation to posterior instrumentation, old techniques such as interfacet wiring have been replaced by lateral mass and pedicle screw fixation systems [32, 33]. The major complications with instrumented fusion are risk of neural injury, adjacent segment degeneration, and vertebral artery injury [8, 34, 35].

#### 5.2.2. Laminoplasty

Laminoplasty has gained more attention in the Japanese literature because of the high prevalence of CSM related to ossification of the posterior longitudinal ligament. The “open-door” technique has been popularized in 1970s by Hirabayashi. Several technical modifications have been proposed throughout the years [34, 36, 37].

Laminoplasty preserves most of the bony posterior vertebral elements and, therefore, may decrease the risk of postlaminectomy kyphotic deformity in comparison with laminectomy. Besides that, in comparison with laminectomy with fusion, laminoplasty seems to present a decreased incidence of adjacent-level degeneration by preserving normal cervical range of motion [34, 36]. 

Although some authors have suggested that cervical fusion (but not laminoplasty) significantly reduces neck pain in patients with stenotic myelopathy [38], we have demonstrated that, up to now, there continues to be no evidence that laminectomy with fusion is better than laminoplasty in reducing neck pain in patients with CSM. In the aforementioned paper, although the reduction in the VAS scores in the laminectomy (but not in the laminoplasty group) was statistically significant (*P* < 0.01), we have shown that there is no study which proves that such reduction reaches the Minimum clinically important difference (MCID) for neck pain in visual analogic scale (VAS) scores [39].

## 6. Conclusions

Cervical spondylotic myelopathy is a prevalent degenerative spinal disease which may lead to significant clinical morbidity. The clinical findings are variable, and both dynamic and static X-rays, as well as MRI, are important for preoperative evaluation as well as individualizing surgical planning. The choice of the most appropriate technique is affected by patient's clinical condition and radiologic findings as well as surgeon's experience.

In opposition to the old belief that patients presenting mild myelopathy should be treated conservatively, there has progressively been amount of evidence indicating that the clinical course of this disease is progressive deterioration and that early surgical intervention improves long-term functional recovery.
